# Losing legs to facilitate sex: *Triramidesmus* gen. nov., a new remarkable monospecific genus of the millipede family Polydesmidae (Diplopoda, Polydesmida) from the Altai Mountains of northeastern Kazakhstan

**DOI:** 10.3897/zookeys.1291.210036

**Published:** 2026-09-04

**Authors:** Pavel S. Nefediev, Sergei I. Golovatch, Julia S. Nefedieva

**Affiliations:** 1 Altai State University, Lenina 61, Barnaul 656049, Russia Altai State University Barnaul Russia; 2 A.N. Severtsov Institute of Ecology and Evolution, Russian Academy of Sciences, Leninsky pr. 33, Moscow 119071, Russia A.N. Severtsov Institute of Ecology and Evolution, Russian Academy of Sciences Moscow Russia; 3 Tomsk State University, Biological Institute, Lenina 36, Tomsk 634050, Russia Tomsk State University, Biological Institute Tomsk Russia

**Keywords:** Iconography, millipede, new genus, new species, sex selection, taxonomy

## Abstract

A new genus and species of Polydesmidae is described from the Altai Mountains of northeastern Kazakhstan: *Triramidesmus kazakhstanus***gen. et sp. nov**. The new genus shows a unique combination of characters which are novel in the entire order Polydesmida: incised or lobulated paraterga, 3-branched gonopod telopodites, the presence of a clear-cut accessory seminal chamber at the base of and inside a short, triangular, spiniform solenomere, and an almost totally reduced leg-pair 2 in the adult female. Presumably only in already mated females, the epigynal aperture is blocked by a subquadrangular, flattened coxae 2, which suggests sexual competition. A distribution map and a detailed iconography using light and scanning electron microscopy are presented.

## Introduction

The Russian Altais is a mountainous territory in the north of Inner Asia (red dotted line in Fig. 7), lying at the junction of four countries, Russia, Kazakhstan, China, and Mongolia, all within the Russian-Altai physiographic region in the Altai-Khangai-Sayan Mountain Country (Chernykh and Zolotov 2011). Being part of the broader Russian Altais, the Kazakh Altais cover the northeastern part of the Republic of Kazakhstan.

The millipede fauna of the Kazakh Altais is very poorly studied, only some 10 species having hitherto been recorded there, as follows.

1. *Orinisobates sibiricus* (Gulička, 1963)

2. *Megaphyllum sjaelandicum* (Meinert, 1868)

3. *Julus kazakhus* Mikhaljova, 2013

4. *Leptoiulus tigirek* Mikhaljova, Nefediev, Nefedieva & Dyachkov, 2015

5. *Sibiriulus multinicus* Mikhaljova, 2001

6. *Altajosoma arshaty* Mikhaljova, 2013

7. *Altajosoma bukhtarma* Mikhaljova, 2013

8. *Polydesmus inconstans* Latzel, 1884

9. *Julus* sp.

10. *Altajosoma* sp.

Of these species, two (9 and 10) are new and await descriptions (Nefediev 2021), whereas one more (8) is an anthropochore introduction (Enghoff 1985; Golovatch 1992; Mikhaljova and Golovatch 2001; Mikhaljova et al. 2013a, 2013b; Nefediev 2018; Nefediev and Nefedieva 2018).

The present paper describes a new monospecific genus of Polydesmidae recently discovered in the Altai Mountains of the Republic of Kazakhstan, Central Asia. However, the assignment of *Triramidesmus* gen. nov. to Polydesmidae is not that straightforward, meriting a discussion.

## Material and methods

The material reported below was taken by Yu.V. Dyachkov (Barnaul, Russia) during his field trip to Kazakhstan in 2024. The specimens are stored in 75% alcohol. The holotype and most of the paratypes are housed in the collection of the Zoological Museum, Lomonosov Moscow State University, Moscow, Russia (**ZMUM**), with only a few duplicate paratypes deposited in the collection of the Altai State University, Barnaul, Russia (**ASU**).

The specimens were examined and measured using a C-mex-10 Pro digital camera attached to a NexiusZoom EVO 1703-S trinocular stereomicroscope, manipulated with Euromex ImageFocusAlpha (v. x64, 1.3.7.15529.20190906) software at the Science Department Laboratory of the Tigirek State Nature Reserve (Barnaul, Russia).

Colour photographs were taken at the Paleontological Institute, Russian Academy of Sciences (PIN), Moscow, using a Flexacam C1 camera mounted on a Leica M165С stereomicroscope with built-in LasX software. Images were processed with Adobe Photoshop CC. Slide-mounted specimens were imaged using an Olympus CX23 microscope equipped with an ADF STD16 camera.

Scanning electron microscopy (SEM) was performed at the Center for Collective Use of Microscopy and X-ray Spectroscopy, Institute for Water and Environmental Problems, Siberian Branch, Russian Academy of Sciences, Barnaul, Russia (IWEP), using a Hitachi S-3400N scanning electron microscope. Mounts for SEM were made by air-drying, mounting on stubs, and coating with gold and palladium (Au/Pd alloy). SEM material was removed from stubs and returned to alcohol after examination. Digital image stacking and final figure plates were processed and assembled in Adobe Photoshop CS6.

The approximate measurements in the images were performed using an online photo measuring tool (https://eleif.net/photomeasure). The distribution map was composed using QGIS v. 4.2.0.

The genital terminology follows that of Golovatch (2014), Golovatch and VandenSpiegel (2015), Djursvoll (2019), Zahnle et al. (2020), Shear and Marek (2021), and Antić et al. (2022), with modifications. The following body ring formula is accepted: **C**ollum + **p**odous rings + **a**podous rings + **T**elson. Abbreviations used to denote habitus and genital structures are explained in the text and figure captions.

## Results

**Class Diplopoda de Blainville in Gervais, 1844**

**Order Polydesmida Pocock, 1887**

**Family Polydesmidae Leach, 1816**

### Genus Triramidesmus gen. nov.

Taxon classificationAnimaliaPolydesmidaPolydesmidae

F9598B2D-1876-5116-9943-92C27C40A731

https://zoobank.org/D95F18D2-CDEA-46A5-BE2D-6FC4E85DEBD7

#### Type species.

*Triramidesmus kazakhstanus* gen. nov. et sp. nov., by monotypy and present designation.

#### Composition.

Monotypic.

#### Name.

The new genus is named after the 3-branched (= triramous) gonopod telopodites, and the suffix *-desmus*, one of the most common suffixes of genera-level taxa in Polydesmida. The name is a noun in masculine gender.

#### Diagnosis.

This genus differs from all other Polydesmida in having an almost totally reduced second leg-pair in adult females, coupled with 3-branched gonopod telopodites, an unusually short seminal groove lying entirely basal to the telopodite branches, the presence of a clear-cut accessory seminal chamber at the base of and partly inside a short, spiniform solenomere, and the absence of a hairy pulvillus terminating the seminal groove.

#### Description.

As for the type species.

#### Affinities.

The new genus and species seem to be especially similar to *Schizoturanius tabescens* (Stuxberg, 1876), a disjunct formal member of the genus *Schizoturanius* Verhoeff, 1931, which is widespread in southwestern Siberia (Fig. 7), with outlying records along the Yenisei River valley in north-central Siberia and isolated reports from the Tyumen and Irkutsk oblasts (Nefediev 2022). Both these taxa share the small body, medium-sized and incised paraterga, but the gonopod conformation is rather simple, with a bipartite acropodite and a long seminal groove in *S. tabescens* (Fig. 5F, J), vs complex, with a tripartite acropodite and a short seminal groove in the new genus and species (Fig. 5E, I).

What seems particularly important is that, based on the gonopod conformation, both *S. tabescens* and *Triramidesmus kazakhstanus* gen. nov. et sp. nov. share a clear-cut accessory seminal chamber, a character typical only of the family Polydesmidae.

Superficially, considering the relatively short seminal groove that fails to move onto an acropodital branch, the presence of a short and simple solenomere, and the absence of a hairy pulvillus on the gonopod exomere can allow us to assign *Triramidesmus* gen. nov. to Trichopolydesmidae*sensu lato* (Antić et al. 2022) rather than Polydesmidae. As a close match, *Triramidesmus* gen. nov. would seem to include the Himalayan trichopolydesmid genus *Hingstonia* Carl, 1935, presently with 13 described, mostly high-montane species (Golovatch 1986, 1988, 1990; Golovatch and Martens 2018). The gonopod telopodite in *Hingstonia* species is 2- to 4-branched, whereas the solenomere is likewise short, simple, and spiniform.

However, the presence alone of a distinct, prominent accessory seminal chamber in *Triramidesmus kazakhstanus* gen. nov. et sp. nov. leaves no doubt that the new genus and species actually belongs to the family Polydesmidae.

### Triramidesmus kazakhstanus sp. nov.

Taxon classificationAnimaliaPolydesmidaPolydesmidae

B637BF24-80DF-56D8-AA41-7A9E5C33FD55

https://zoobank.org/8A94A1EC-47BC-4BCD-8ACD-0CD5FC553227

**Figure 1.**
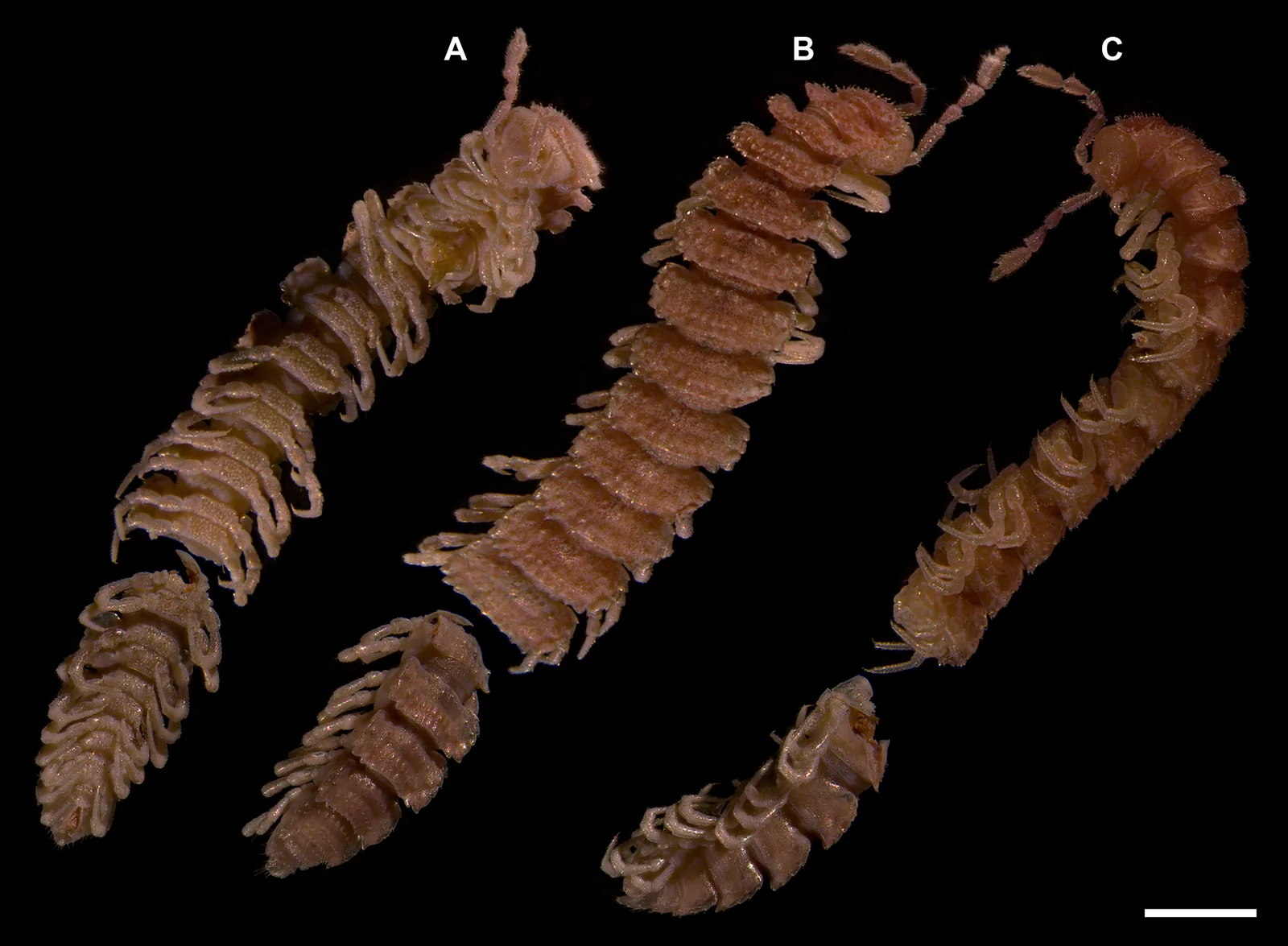
*Triramidesmus kazakhstanus* gen. nov. et sp. nov., ♂ paratype (ZMUM), habitus. **A–C**. Ventral, dorsal, and lateral views, respectively. Scale bar: 1 cm.

**Figure 2.**
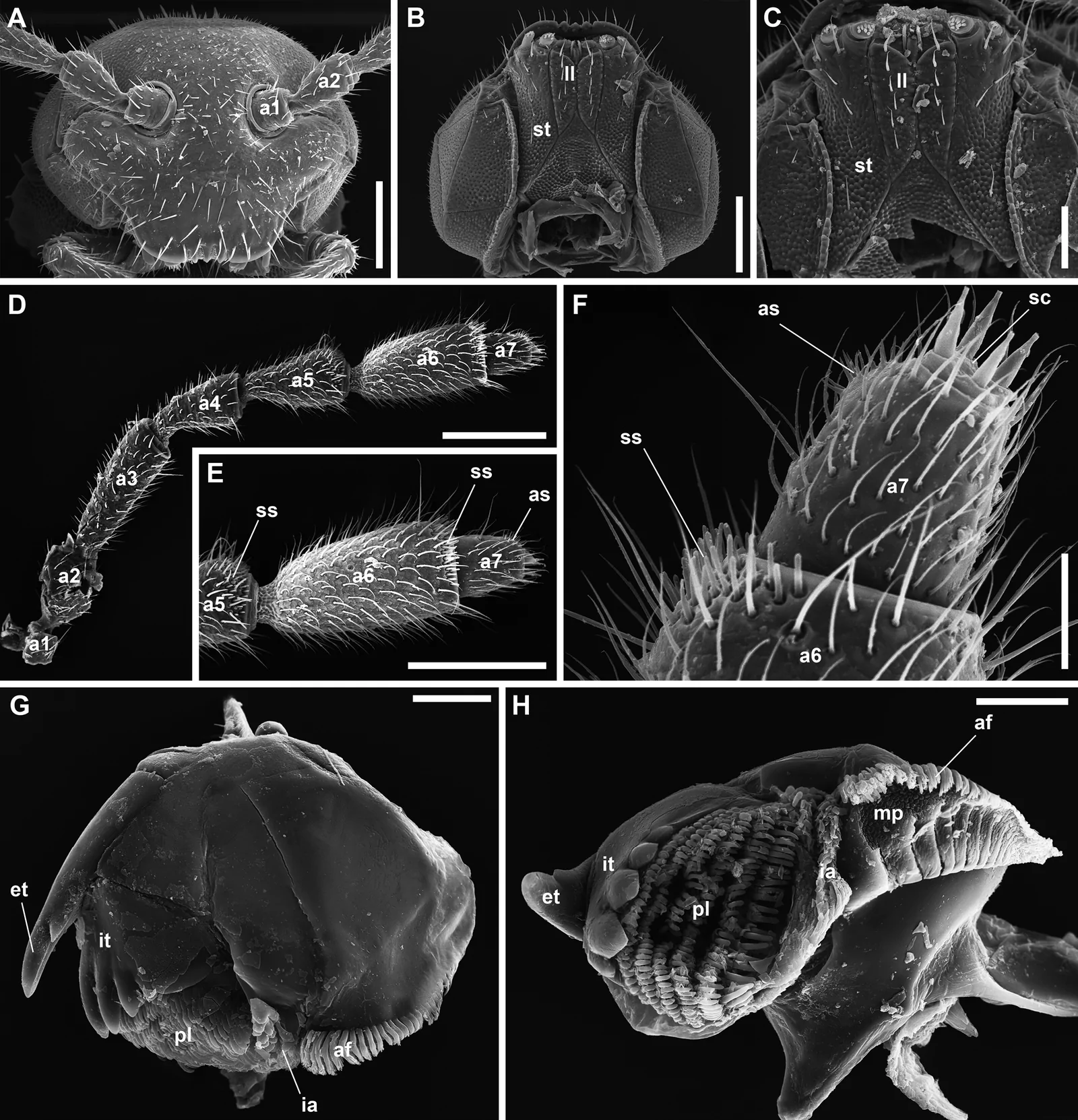
*Triramidesmus kazakhstanus* gen. nov. et sp. nov. **A**, **C**. Paratype ♀ (ASU); **B**, **D–H**. Paratype ♂♂ (ASU); **A**. Head, anterior view; **B**. Head, ventral view; **C**. Ghathochilarium, ventral view; **D**. Antenna, lateral view. **E**, **F**. Distal part of antenna, lateral view. **G**, **H**. Gnathal lobe, lateral and anterior views, respectively. Abbreviations: **a1–a7** antennomeres 1–7, respectively, **af** anterior fringe of mandibular gnathal lobe, **as** acuminate sensilla on antennomere 7, **et** external tooth of mandibular gnathal lobe, **ia** intermediate area of mandibular gnathal lobe, **it** internal tooth of mandibular gnathal lobe, **ll** lamella lingualis, **mp** molar plate of mandibular gnathal lobe, **pl** pectinate lamella of mandibular gnathal lobe, **sc** sensory cones on terminal disc of antennomere 7, **ss** subbacilliform sensilla on antennomeres 5 and 6, **st** stipes. Scale bars: 200 µm (**A**, **B**, **D**, **E**); 50 µm (**C**, **F**, **G**, **H**).

**Figure 3.**
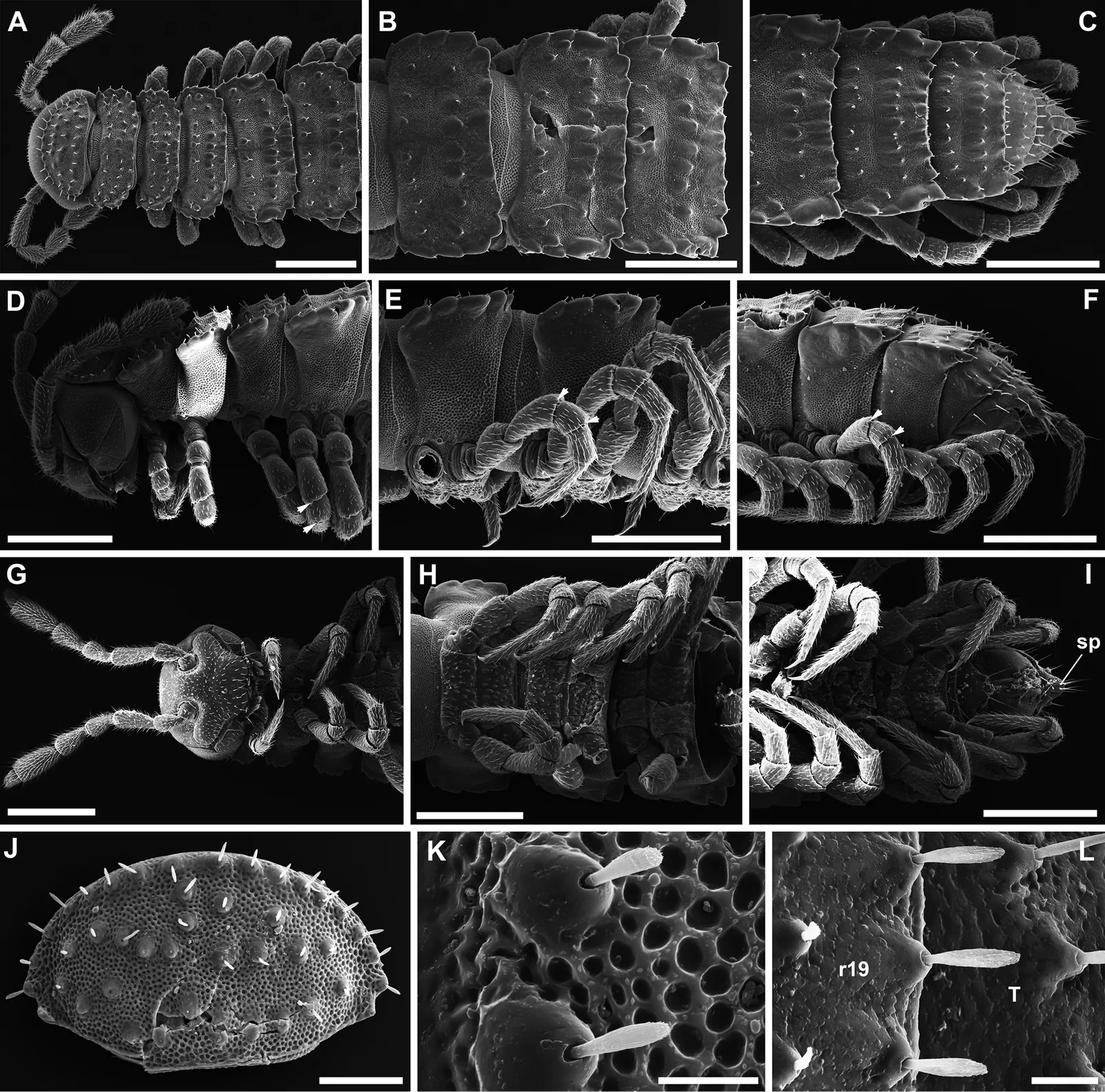
*Triramidesmus kazakhstanus* gen. nov. et sp. nov., paratype ♂♂ (ASU), habitus. **A, D, G**. Anterior part of body, dorsal, lateral, and ventral views, respectively; **B, E, H**. Midbody rings, dorsal, lateral, and ventral views, respectively; **C, F, I**. Posterior part of body, dorsal, lateral, and ventral views, respectively; **J**. Collum, dorsal view; **K**. Haplotergal setae on ring 4, dorsal view; **L**. Metatergal setae on ring 19, dorsal view. Abbreviations: **r19** ring 19, **sp** spinnerets on epiproct, **T** telson, arrows show distodorsal protrusions. Scale bars: 500 µm (**A–I**); 150 µm (**J**); 30 µm (**K**, **L**).

**Figure 4.**
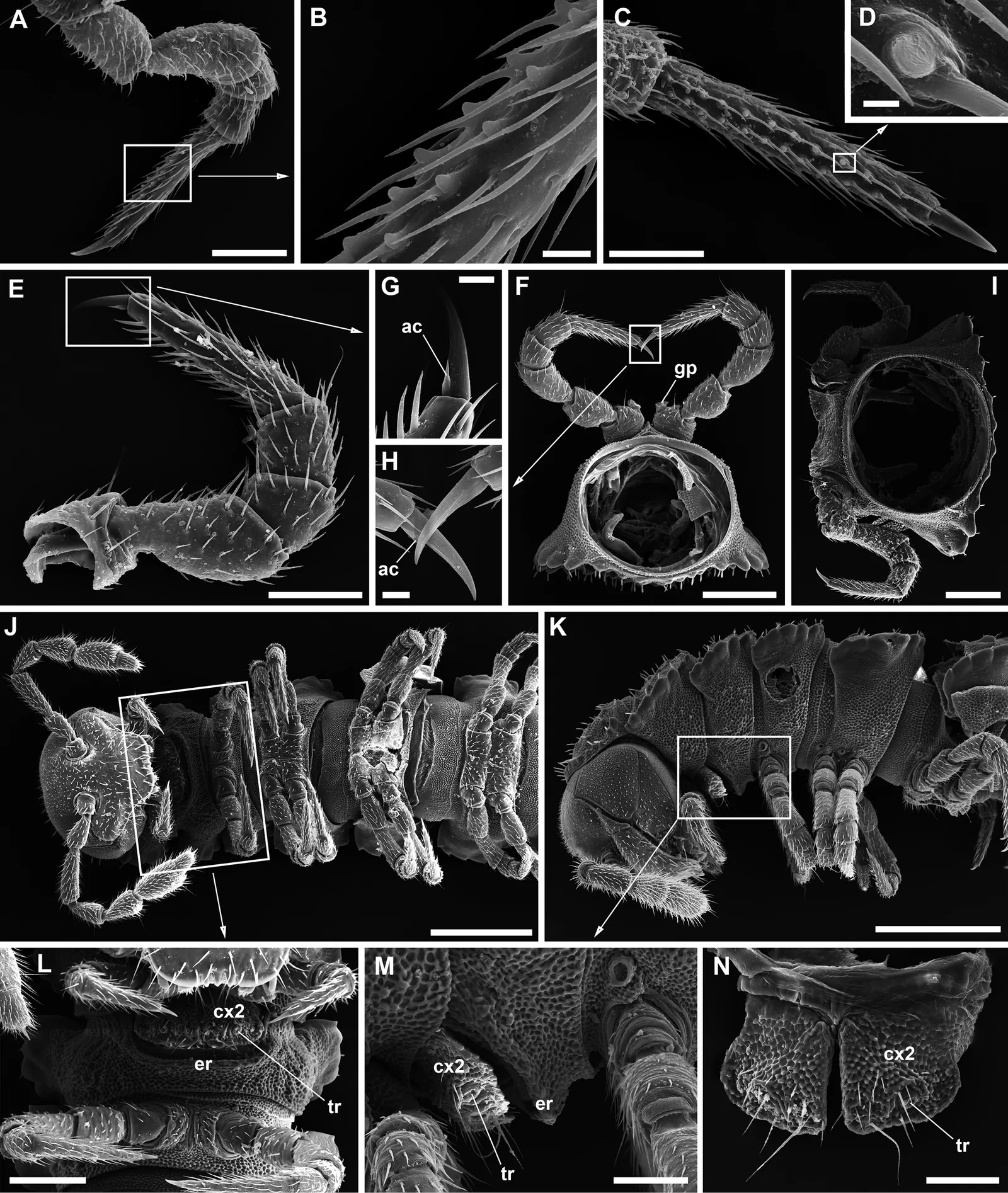
*Triramidesmus kazakhstanus* gen. nov. et sp. nov. **A–I**. Paratype ♂♂ (ASU); **J–N**. Paratype ♀ (ASU); **A**. Leg 9, lateroventral view; **B**. Sphaerotrichomes on tarsus 9, lateroventral view; **C**. Tarsus 9, ventral view; **D**. Sphaerotrichome on tarsus 9, ventral view; **E**. Leg 1, lateral view; **F**. Ring 3 with leg-pair 2, posterior view; **G**. Distal part of tarsus 1, lateral view; **H**. Distal part of tarsi 2, lateral view; **I**. Ring 7 with leg-pair 9, posterior view; **J, L**. Anterior body part, ventral view; **K, M**. Anterior body part, lateral view; **N**. Coxae 2, ventral view. Abbreviations: **ac** accessory claw, **cx2** coxa 2, **er** epigynal ridge, **gp** gonapophysis, **tr** telopodite remnant of ♀ leg-pair 2. Scale bars: 150 µm (**A**); 20 µm (**B**, **G**, **H**); 100 µm (**C**, **E**, **M**, **N**); 5 µm (**D**); 250 µm (**F**, **I**); 500 µm (**J**, **K**); 200 µm (**L**).

**Figure 5.**
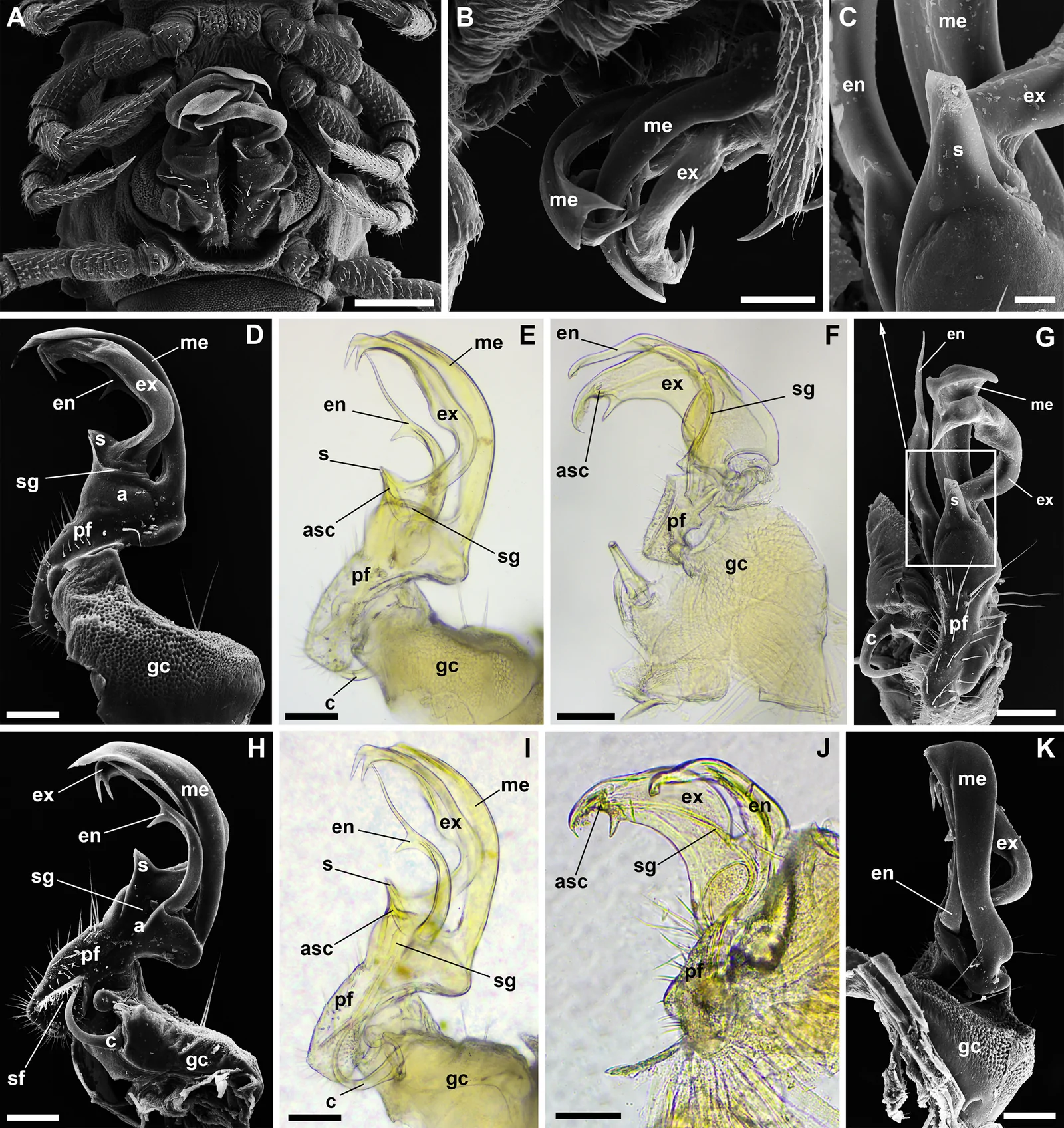
*Triramidesmus kazakhstanus* gen. nov. et sp. nov. **A–D, G, H, K**. Paratype ♂♂ (ASU); **E, I**. Paratype ♂ (ZMUM), *Schizoturanius tabescens*; **F, J**. ♂ (ZMUM); **A, B**. Gonopods *in situ*, ventral and lateral views, respectively; **C**. Basal part of gonopod acropodite, posterior view; **D, E**. Gonopod, lateral view; **F, J**. Gonopod, lateral and mesal views, respectively; **G, K** Gonopod, posterior and anterior views, respectively; **H, I**. Gonopod, mesal view. Abbreviations: **a** acropodite, **asc** accessory seminal chamber, **c** cannula, **en** gonopod endomere, **ex** gonopod exomere, **gc** gonocoxite, **me** gonopod mesomere, **pf** prefemorite, **s** solenomere, **sf** seminal fossa, **sg** seminal groove. Scale bars: 250 µm (**A**); 100 µm (**B**, **D**–**K**); 25 µm (**C**).

**Figure 6.**
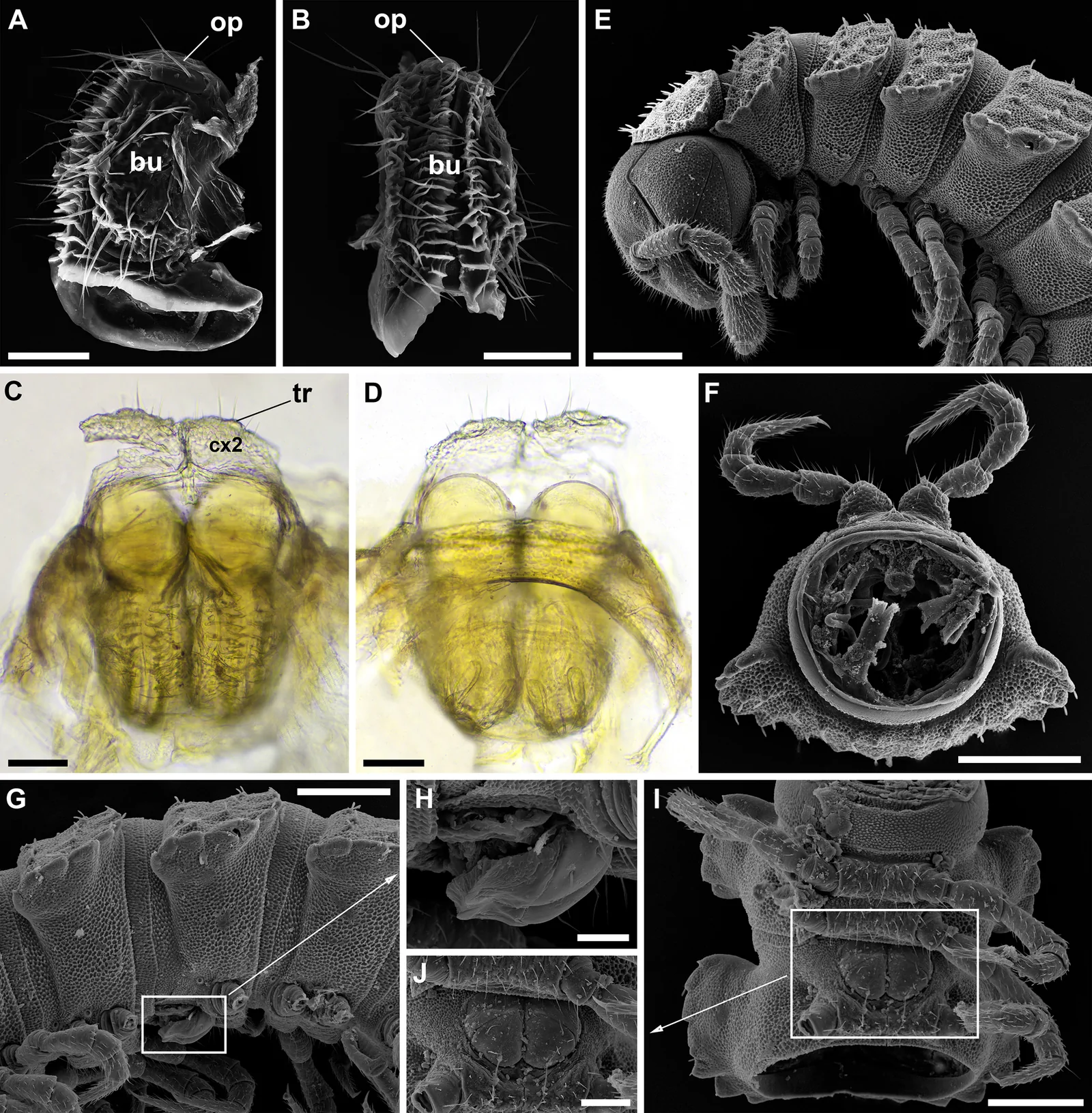
*Triramidesmus kazakhstanus* gen. nov. et sp. nov. **A**, **B**. Paratype ♀ (ASU); **C, D**. Paratype ♀ (ZMUM); **E, F**. Paratype subadult ♀♀ (ASU); **G–J**. Paratype subadult ♂♂ (ASU); **A**. Vulva, lateral view **B**. Vulva, anterior view; **C, D**. Both vulvae *in situ*, anterior and posterior views, respectively; **E**. Anterior body part, lateral view; **F**. Ring 3 with leg-pair 2, anterior view; **G**. Rings 6–8, lateral view; **H**, **J**. Primordia, lateral and ventral views, respectively **I** rings 6–7, ventral view. Abbreviations: **bu** bursa of vulva, **cx2** coxa 2, **op** operculum of vulva, **tr** telopodite remnant of ♀ leg-pair 2. Scale bars: 100 µm (**A**–**D**, **J**); 250 µm (**E**–**G**); 50 µm (**H**); 350 µm (**I**).

Figs 1, 2, 3, 4, 5, 6

#### Material examined.

***Holotype*** ♂ (ZMUM, Rd 5591): Kazakhstan, East Kazakhstan Region [= Oblast], Ulan District, 10 km SE of Verkhnie [not Nizhnie as noted on label] Tainty Village, Kalbinskiy Mt. Range, 49°19'11.46"N, 83°06'41.41"E [= 49.3199, 83.1115], *Populus*, *Betula*, and *Salix* forest [with tall grass vegetation], in litter and soil, 1120 m, 16.VIII.2024, Yu.V. Dyachkov leg.

***Paratypes***. 30 ♂♂, 1 subadult ♂, 2 subadult ♀♀ (ZMUM, Rd 5592), 1 ♀ (ZMUM, Rd 5593), 10 ♂♂, 1 ♀, 2 subadult ♂♂, 2 subadult ♀♀ (ASU.NPS.D-031), same data as for holotype.

#### Name.

To emphasise the provenance of the new species from Kazakhstan. Adjective.

#### Diagnosis.

As for the monospecific genus.

#### Description.

Body moniliform, with 20 rings (C+17p+1a+T) in adults of both sexes. Holotype ♂, 11.4 mm long, width of midbody pro- and metazona 0.75 and 1.1 mm, respectively. Paratype ♂♂, 11.4–12.1 mm long, width of midbody pro- and metazona 0.75 and 1.1 mm, respectively. Paratype ♀, 11.1 mm long, width of midbody pro- and metazona 0.75 and 1.1 mm, respectively. Paratype subadult specimens at stadium VII (19 rings: C+16p+1a+T), 7.9–8.6 mm long, 0.6 and 0.9 mm wide on midbody pro- and metazona, respectively.

Colouration in alcohol pinkish beige, legs lighter (Fig. 1A–C). Tegument moderately shining throughout; texture very delicately shagreened, finely alveolate laterally and dorsally.

Head mostly densely pubescent, with convex genae; clypeolabral region stretched forward, with stronger setae, but occiput and postantennal regions bare (Figs 2A, 3G). Antennae moderately long, clavate, *in situ* reaching past ring 2 dorsally; in length, antennomere 3 > 6 > 5 > 4 > 2 > 7 > 1; antennomeres 5 and 6 (**a5** and **a6**) each with a small group of subbacilliform sensilla (**ss**) distodorsally (Fig. 2D, E), neither drawn inside a pit; antennomere 7 (**a7**) with a terminal disc bearing four sensory cones (**sc**), and a small, distodorsal bunch of a few acuminate sensilla (**as**) (Fig. 2F). Mandibular gnathal lobe: external tooth (**et**) with a large apical cusp and one smaller subapical cusp laterally; internal tooth (**it**) comb-shaped, with five apically pointed cusps; six rows of pectinate lamellae (**pl**) with simple, bacilliform teeth; intermediate area (**ia**) with serrate papillae; molar plate (**mp**) microsculptured, with transverse ridges, its anterior fringe (**af**) with serrate spines apically (Fig. 2G, H). Genae squarish, gnathochilarium typical of Polydesmida, each stipes (**st**) with 10–12 setae (♂) or 7–10 setae (♀), each lamella lingualis (**ll**) with seven or eight setae (♂) or six setae (♀), these increasingly long distally and regularly arranged in a line (Fig. 2B, C).

In width, collum < head (0.8 mm broad) = ring 2 = 3 < 4 < 5 < 6 < 7 = 16; thereafter body gradually tapering towards ring 18, then significantly tapering on ring 19 and telson (0.3 mm broad) in both sexes. Collum (Fig. 3J) transversely ellipsoid, anteriorly semicircular, with 8+9 setae along anterior edge; with one incision laterally; three more or less regular rows of medium-sized, fusiform, longitudinally ribbed setae: 2+2 – 3+4 setae in first row, 2+4 setae in second row, 5+6 setae in third row, while following metaterga with three regular rows of 3+5 – 6+7 setae each. Haplotergal surfaces of rings 2–4 increasingly short compared to following ones, barely convex or almost flat (Fig. 3A). Dorsal surface of collum, tergites on haplo- and diplosegments (metatergites) macrosculptured. Metatergal polygonal sculpture (bosses) missing, replaced by three transverse rows of distinct minute knobs or rounded tubercles, more distinct on a few anterior most rings (Fig. 3A–C); each minute knob or tubercle supporting a simple, short, fusiform, longitudinally ribbed tergal seta at its top or posterior margin (Fig. 3K, L); telson with long, conical setae arranged in irregular rows. Paraterga moderately developed, set high, with thin calluses, starting with postcollum rings, subhorizontal, but always lying slightly below a faintly convex dorsum (Fig. 3A–F); anterolateral and posterolateral corners of postcollum paraterga increasingly pointed posteriorly and extending beyond rear tergal margin, especially clearly so in segments 17 and 18 (Fig. 3C). Lateral edges of slightly swollen paraterga incised, smooth, with four or five setigerous lobules (Fig. 3A, B). Pore formula normal: 5, 7, 9, 10, 12, 13, 15–18; 4+4 fusiform setae on poreless rings and 5+5 setae on poriferous ones; poriferous paraterga with enlarged penultimate lateral lobule bearing a teardrop-shaped ozopore (Fig. 3D, E). Limbus very fine, delicately and sparsely microdenticulate. Epiproct rather long, rounded at tip, directed slightly caudoventrad, carrying two couples of relatively long, acuminate, dorsal and lateral setae, ending with typical four elongated, setiform spinnerets (**sp**) (Fig. 3I). Paraprocts semispherical, each with two long setae originating from small tubercles. Hypoproct trapeziform, with two long distal setae also originating from minute knobs (Fig. 3I).

Legs generally rather long and slender, slightly incrassate, and longer in ♂ compared to ♀. In ♂, prefemora, femora, postfemora, and tibiae somewhat dorsally swollen; prefemora especially well bulging; leg-pair 1 smaller compared to following walking legs; tarsi 2 and following ones ventrally with one or two rows of sphaerotrichomes (Fig. 4A–D). In both sexes, podomeres setose; coxae and prefemora each distoventrally with a long, medial macroseta; tibiae each distodorsally with a long, medial macroseta (Fig. 4F); femora and postfemora each with a prominent distodorsal protrusion (Fig. 3D–F); tarsi 1 and 2 longest, slender, with robust dense setae ventrally, each claw slightly curved, with an accessory claw (**ac**) anteroventrally (Fig. 4E–H). ♂ coxae 2 with very short, membranous, cylindrical gonapophyses (**gp**) (Fig. 4F). ♀ leg-pair 2 reduced to telopodite remnants (**tr**) on subquadrangular flattened coxae 2 (**cx2**), these covering vulvar aperture (Fig. 4J–N). ♂ pregonopodal sternite of leg-pair 5 with minute medial incision, while following sternite of leg-pair 6 reduced, concave inside, without setae, creating a gap in which gonopods lie when retracted (Fig. 5A). Postgonopodal sternite of leg-pair 9 compressed frontocaudally, flat, bent posteriad, with minute, simple setae (Figs 4I, 5A).

Gonopod aperture transversely oblong-oval, taking up most of ventral part of metazonite 7, with an elevated caudal margin (Fig. 5A). Gonopods (Fig. 5A–E, G–I, K) complex, each with a subquadrate and microgranulate gonocoxite (**gc**) laterally; **gc** carrying two macrosetae on ventral face and a normal, C-shaped cannula (**c**) mesally (Fig. 5D, G, H). Telopodite almost twice as long as **gc**, distal end strongly curved laterally. Prefemorite (**pf**) densely setose as usual, coaxial with body, but subrectangular to acropodite (**a**); seminal fossa (**sf**) with two folds, both setose from inside (Fig. 5H). Acropodite (**a**) mostly bare (Fig. 5D, H), divided longitudinally into three subfalcate branches (Fig. 5D–E, G–I, K): (1) exomere (**ex**), the lateral branch curved ventrad, then bent laterad at a nearly right angle, ventrobasally with a bifid apex and a stout, triangular, dentiform, subtruncate solenomere (**s**) (Fig. 5C); (2) mesomere (**me**), the median branch, sigmoid at base, somewhat broadened near an elongate apex; and (3) endomere (**en**); mesal branch, slender, with a pointed midway ventral process and a long subulate apex. Seminal groove (**sg**) relatively short, lying fully basal to all three branches of **a**, starting from **sf** mesally on **pf**, first running along mesal side of **a** (Fig. 5H, I) to form a distinct loop laterad at bases of and between two acropodital branches, **me** and **ex**, to finally terminate laterally inside a prominent, elongated accessory seminal chamber (**asc**, = ampulla, = vesicle) on **s** (Fig. 5D, E). Hairy pulvillus near tip of **s** absent.

In ♀, a transverse epigynal ridge (**er**) behind vulvar aperture well developed, subrectangular, transverse, and straight, without a median outgrowth (Fig. 4L). Vulvae bean-shaped, *in situ* fully blocked by subquadrangular flattened coxae 2 (**cx2**), these latter bearing telopodite remnants of legs 2. Anterior surface of bursa (**bu**) covered with two rows of relatively large apodematic projections of spermatheca, laterally with 10 pairs of setiform fimbriae along these projections, some twisted at end, starting on top and following 2/3 of vulva extent (Fig. 6A, B); lateral surfaces of **bu** with robust, long, acuminate setae; operculum (**op**) with four pairs of long setae. Sac- or spiral-shaped structures present at bottom of bursa’s gutter (Fig. 6C, D).

In immature specimens of stadium VII (body with 19 rings), each subadult ♂ with a pair of tiny rounded gonopodal primordia (Fig. 6I, J) or elongated, but clearly underdeveloped gonopods (Fig. 6G, H) instead of leg-pair 8. Subadult ♀♀ with normal walking leg-pairs 2 (Fig. 6E, F).

#### Distribution.

**Figure 7.**
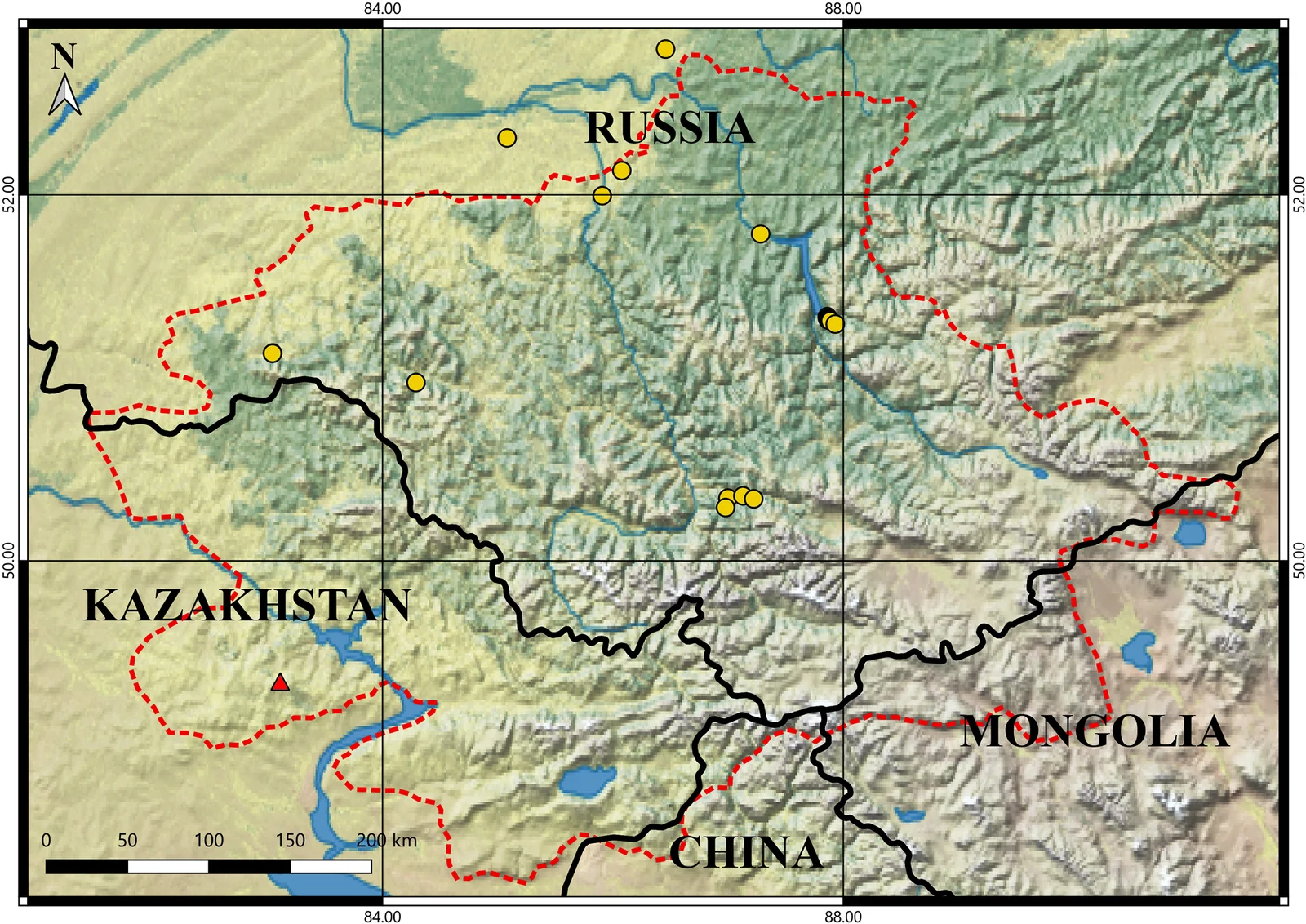
Distributions of *Triramidesmus kazakhstanus* gen. nov. et sp. nov. (red triangle), and *Schizoturanius tabescens* (yellow circle) within the Russian Altais (red-dotted line).

So far, known only from its type locality, the Kalba Mt. Range, East Kazakhstan Oblast, Kazakhstan (Fig. 7).

#### Habitats.

**Figure 8.**
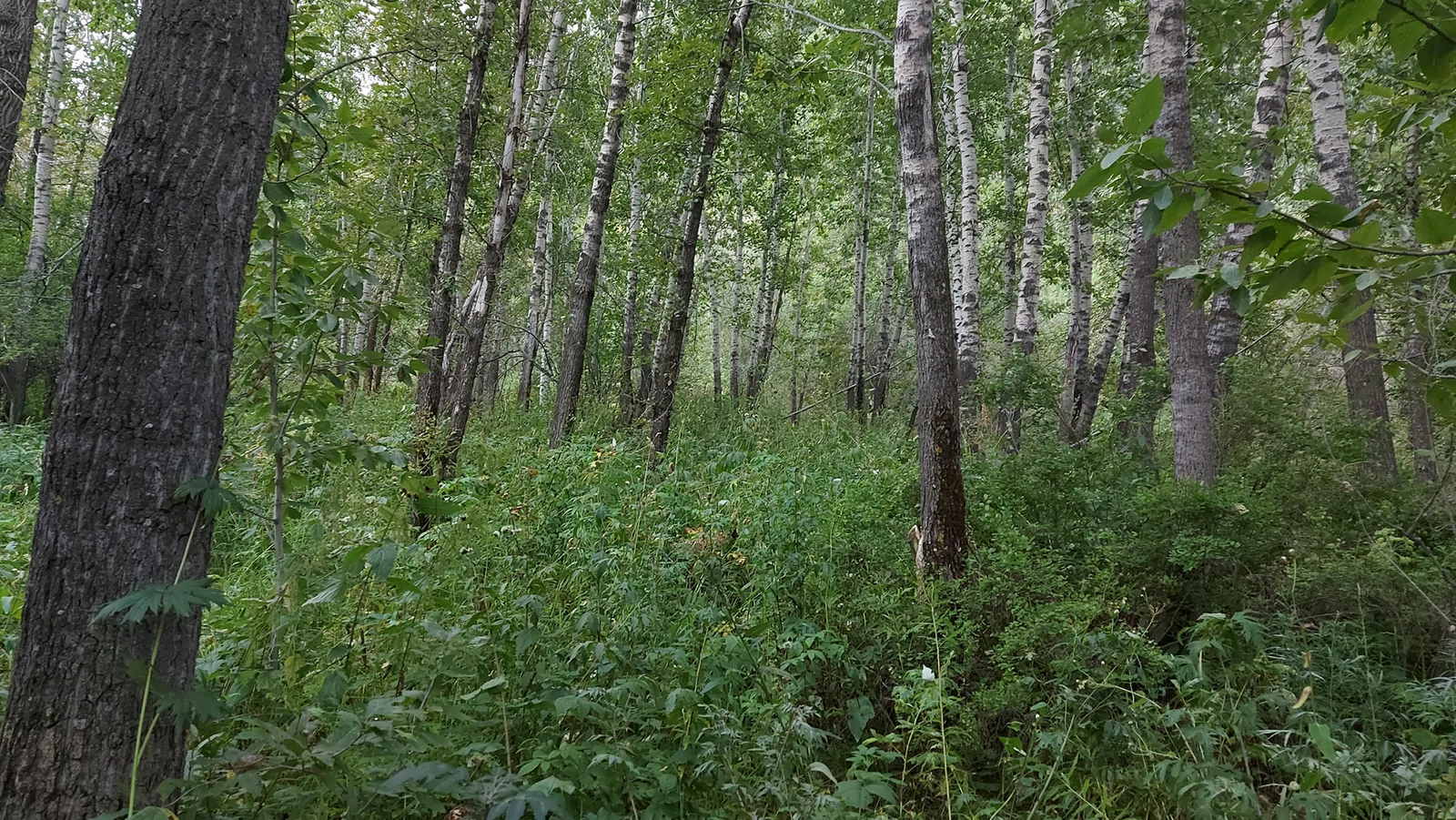
Habitat of *Triramidesmus kazakhstanus* gen. nov. et sp. nov. (courtesy Yu.V. Dyachkov).

The new species was collected in the mid-montane belt of the Altais in a small-leaved forest with *Populus*, *Betula*, and *Salix* (Fig. 8) at 1120 m a.s.l., dwelling in leaf litter and the upper soil layer.

#### Remarks.

The strikingly reduced female leg-pair 2 of the new genus and species appears to be unique in the entire order Polydesmida. However, the reduction of the second pair of female legs to tiny vestiges is well known in several orders of Diplopoda, i.e. Chordeumatida (Mastigophorophyllidae Verhoeff, 1899, *Ghilarovia* Gulička, 1972, *Kashmireuma* Mauriès, 1982), Julida, Blaniulidae C.L. Koch, 1847 (*Archichoneiulus* Brolemann, 1921, *Microchoneiulus* Brolemann, 1921, *Nopoiulus* Menge, 1851), Callipodida (partly *Bollmania* Silvestri, 1896, *Callipus* Risso, 1826, *Cyphocallipus* Verhoeff, 1909, *Euxinopetalum* Hoffman, 1973, *Paracortina* Wang & Zhang, 1993) (Enghoff 1984; Mauriès 1988; Mikhaljova 2002; Stoev and Enghoff 2005; Minelli 2015).

#### Discussion.

The accepted genital terminology in the Polydesmoidea is often too complicated, confusing, and unclear, and this apparently reflects the usually complex structure of the gonopods and vulvae in Polydesmida. The same terms are often used to designate structures that are evolutionarily barely related, especially gonopodal conformations (see Golovatch 2014; Golovatch and VandenSpiegel 2015; Djursvoll 2019; Zahnle et al. 2020; Shear and Marek 2021; Antić et al. 2022). To avoid confusion, we apply a strictly topological approach to the branches of the gonopodal acropodite instead of a homology-based evolutionary one, proposing the following terminology. The lateral branch is to be termed the exomere (the prefix “exo-” derives from the Greek “ἔξω”, and means “outside”, the suffix “mere” derives from the Greek “μέρος”, meaning “a part”). The inner branch is to be termed the endomere (the prefix “endo-” derives from the Greek “ἔνδον” and means “inside”). Finally, the median branch lying between exo- and endomere is to be designated the mesomere (the prefix “meso-” derives from the Greek “μέσος” and means “median”).

*Triramidesmus kazakhstanus* gen. et sp. nov., in having the female leg-pair 2 almost totally reduced, is remarkable in only minute remnants of telopodites 2 being traceable in the middle of subquadrangular, flattened coxae 2 in adult females. Apparently, this reduction is the norm for this species, being neither a teratological nor an aberration. This is attested by both known adult females.

In females of three species of *Tasmaniosoma* Verhoeff, 1936, of the polydesmidan family Dalodesmidae, legs 2 are usually missing (Mesibov 2010), seemingly removed by males during copulation, broken off, and “healed”. The male can be assumed to find it easier to copulate with those legs gone (R.E. Mesibov pers. comm.). If so, then *Triramidesmus kazakhstanus* gen. et sp. nov. takes one step further, with the loss of certain legs (female leg-pair 2) apparently serving to facilitate copulation.

The presence of a normal walking leg-pair 2 in 19-segmented subadult females indicates that leg reduction occurs during the last molt from stadium VII (subadult) to stadium VIII (adult). Moreover, as it is impossible to admit that the epigynal aperture is always closed, rendering the vulvae always being blocked and thus not available for mating, the two adult females in the type series, both showing blocked genital apertures and complex vulvae, all definitely functional, must have undergone copulation. It is possible that the male during mating has closed the aperture with flattened coxae 2. Such a situation strongly suggests sexual competition.

That the total collection of the new species amounts to 40+ males and only two females seems quite strange and interesting. In order not to breed speculation, it is necessary to conduct additional research.

## Supplementary Material

XML Treatment for Triramidesmus

XML Treatment for Triramidesmus kazakhstanus

## Additional information

### Artificial Intelligence (AI) use

The authors accept full responsibility for the content of the manuscript, including the disclosure of any use of AI.

No AI tools were used in the preparation of this manuscript.

### Author ORCIDs

P.S. Nefediev https://orcid.org/0000-0001-6074-5635

J.S. Nefedieva https://orcid.org/0000-0002-2501-3698

### Data availability

All of the data that support the findings of this study are available in the main text.
